# Socio-Economic Disparities in Access to Diagnostic Neuroimaging Services in the United Kingdom: A Systematic Review

**DOI:** 10.3390/ijerph182010633

**Published:** 2021-10-11

**Authors:** Aleesha Karia, Reza Zamani, Mohammad Akrami

**Affiliations:** 1 Medical School, College of Medicine and Health, University of Exeter, Exeter EX1 2LU, UK; ak664@exeter.ac.uk (A.K.); r.zamani@exeter.ac.uk (R.Z.); 2 Department of Engineering, College of Engineering, Mathematics and Physical Sciences, University of Exeter, Exeter EX4 4QF, UK

**Keywords:** neuroimaging, health inequalities, BAME (Black, Asian and Minority Ethnic), computed tomography, magnetic resonance imaging, single-photon emission computed tomography, ultrasonography

## Abstract

Socio-economic factors affecting health care can lead to delays in diagnosis of neurological conditions, consequentially affecting treatment and morbidity rates. This inequality in health care can leave patients from lower socio-economic backgrounds more vulnerable to a poorer quality of care from health care providers in the United Kingdom (U.K.). Aims: In this systematic review, we assess the impact of socio-economic status on the use of diagnostic neuroimaging in the U.K., measured by the timeliness, accessibility and appropriate use of computed tomography (CT), magnetic resonance imaging (MRI), ultrasonography, electroencephalography (EEG) and single-photon emission computed tomography (SPECT). We specifically evaluate the non-surgical use of neuroimaging techniques as this relies on the judgment of primary care-givers (e.g., doctors and radiologists), where health disparities are most common. This study includes the analysis of diagnostic imaging used for dementia, minor head injury, stroke, cancer, epilepsy, chronic inflammatory demyelinating polyneuropathy and Parkinson’s disease. With this study, we aim to assess the health inequalities at disease diagnosis. Methods: Using Medline (via Ovid), PubMed and Web of Science databases as sources of information, we critically appraise existing studies on neuroimaging use in the U.K. health care system, published between January 2010 and February 2021. Findings: A total of 18 studies were included in this research, revealing that there was an increase in patients of Black and Asian communities diagnosed with dementia and at an earlier age. There was little evidence to suggest that a lack of access to diagnostic imaging is associated with socio-economic status. However, there are data to suggest that people of a lower socio-economic background require more specialist services with diagnostic neuroimaging tools. In addition, there is evidence to suggest that diagnostic neuroimaging techniques could be utilised more effectively by health care workers to prevent unnecessary delays in diagnosis for patients in lower socio-economic areas.

## 1. Introduction

### 1.1. Rationale

Neurological conditions can progress rapidly, so an early diagnosis is vital for treatment and survival. It is recognised by the National Health Service (NHS) that delays in primary and secondary care can lead to a delayed diagnosis, which could have major consequences for treatment and morbidity rates [1]. In recent years, the knowledge and awareness surrounding health inequalities have increased. The complex dynamics of the social determinants has an effect on the timeliness of diagnosis and, consequently, disease progression [2,3]. In the more diverse and aging population, people are living longer and the demographic of patients is continually changing. This means that the prevalence of neurological diseases is projected to increase alongside the diversity of the aging population within 10 years [4,5,6,7,8].

Socio-economic status historically categorises social groups into race which is reflected in the current policies, practices and services affecting health care [9]. Hence, it is critical to identify the specific aspects of health care that are imbalanced to suggest improvements that could support Black, Asian and Minority Ethnic (BAME) communities. Whilst primary care interventions were introduced to increase accessibility to health care, it is reported that general practitioners (GP) can act as gate keepers to intervention, causing delays in diagnosis and treatment [10]. Early diagnosis of neuro-disease or trauma is essential to reduce the risk of morbidity and mortality of patients. Neuro-disease can be degenerative, such as dementia, with limited treatment options available, meaning that early diagnosis will allow patients the opportunity prepare and plan their care while they still have the mental capacity to make decisions [11]. In cases such as tumour growth and minor head injury, a timely diagnosis would benefit the likelihood of survival and prevent further neurological damage that could affect the patient’s quality of life. 

A systematic review on the utilisation of the neurological care system was conducted in the United States of America (USA), which concluded that there are inequalities in access to health care in neurology. The implications of this observed a lack of engagement from minority communities at low-level treatments and routine appointments. Consequently, this study reported an overrepresentation of minority communities seeking emergency care for severe illness. This is not only more costly for the hospital and patient, but there are greater risks associated with delayed treatment [12]. This research emphasises the importance of early care and diagnosis to support patients and allow them to make informed decisions on their care before they lose autonomy over their own health care. It is suggested that the disparities within neurologic health care was multifactorial and affected by socio-economic status of patients and a lack of representation in neurology. 

As highlighted by the Black Lives Matter movement, racial disparities and health inequalities in the U.K. are similar to America; therefore, it would be informative to see if these findings are replicated [13]. Unlike the USA, the U.K. has a public health care system that intends to mitigate the economic disparities in diagnostic imaging. However, there are additional socio-economic barriers, other than financial burden, which may affect accessibility. 

This review specifically focusses on highlighting the barriers to timeliness, accessibility and appropriate use of diagnostic neuroimaging for diseases or conditions as this would indirectly affect the patient’s treatment and morbidity. 

Table 1 provides an overview of current imaging techniques used for the diagnosis of dementia, minor head injury (MHI), stroke, cancer and epilepsy. Table 2 summarises the reasons why computed tomography (CT), magnetic resonance imaging (MRI), ultrasound, electroencephalography (EEG) and single-photon emission computed tomography (SPECT) may be utilised in diagnosis.

**Table 1:** Overview of the use of diagnostic neuroimaging techniques in dementia, minor head injury, stroke, cancer, epilepsy, CIDP and Parkinson’s disease. This table describes the type of neuroimaging technology recommended for use in the U.K. for the diagnosis of dementia, MHI, stroke, cancer, epilepsy, CIDP and Parkinson’s disease. Recommendations are based on NHS and NICE guidelines. Information was sourced from various resources [14,15,16,17,18,19,20,21]. Refer to the list of abbreviations.

Head or Brain Condition	Diagnostic Imaging Use
Dementia	Patient history and cognition assessments are taken at a primary care service, often a local GP. They will then refer patients to a specialist service, such a Memory Assessment Service (MAS) clinic, where further cognition exams and brain imaging may be taken with either CT or MRI to determine the sub-type of dementia. Additional imaging tools such as PET, SPECT or EEG may be performed to rule out any tumours or fluid-build up or create a higher-resolution image.
Minor head injury (MHI)	Usually, a CT scan is the most efficient and cost-effective approach to determine the structure of the brain. MRI provides a higher-resolution image of the tissue.
Stroke	CT or MRI scans are used to classify whether the stroke is ischaemic or haemorrhagic to identify the treatment pathway and prevent further damage. The National Stroke Strategy in England advises that patients receive brain imaging immediately within normal working hours and within 60 min of out-of-hours service.
Cancer	The “two-week rule” on oral cancer requires that all potential cases of cancer be assessed by secondary specialist care within 14 days after the referral from primary care. Imaging may consist of an endoscopy, X-ray, CT, MRI or PET. In addition to diagnostic imaging, a biopsy may be performed.
Epilepsy	EEG should be performed within 4 weeks of request by a professional. This will support the diagnosis from clinical presentation. MRI should be the imaging of choice for the diagnosis of epilepsy to identify gross pathology.
Parkinson’s Disease	SPECT may be performed to differentiate tremors as PD.
Chronic Inflammatory Demyelinating Polyneuropathy (CIDP)	MRI imaging is used to confirm inflammation of nerve roots with the addition of nerve condition tests and electromyography.

**Table 2:** Summary of diagnostic neuroimaging techniques.

Type of Neuroimaging	Description	Advantages	Disadvantages
Computed Tomography (CT)	Measures the absorption of X-rays Show the density of structures	Efficient Cost-effective	Low resolution of gross structures Higher levels of radiation
Magnetic Resonance Imaging (MRI)	Changes in electrically charged molecules in a magnetic field More precise than X-ray	Non-invasive Very few health risks associated	Patient must keep still
Diagnostic Ultrasonography (Ultrasound)	Ultrasonic waves to image internal body tissue structure	Differentiates diseased arteries Cheap Non-invasive	Sensitivity of imaging is variable
Electroencephalography (EEG)	Records electrical waves Detects abnormal activity (e.g., seizures and sleep disorders	Non-invasive Detects changes on a millisecond level High temporal resolution	Low spatial resolution compared to MRI Portable Less training required
Single-photon emission computerised tomography (SPECT)	Nuclear imaging technique that measures blood flow in the brain to differentiate active and non-active areas	Nuclear imaging does not expose the patient to additional radiation	Expensive equipment and therefore less available Radioactive tracer may cause bleeding, pain or trigger allergies

This table describes the reasons particular imaging techniques may be used or suggested for use. Information sourced from various sources [22,23,24,25].

### 1.2. Objectives

The aim of this study is to examine the characteristics of patients undergoing diagnostic neuroimaging across the U.K., including race, socio-economic status and disease severity, to determine whether there is a significant inequality in access to the required neuroimaging technology. As quality of health care varies across subpopulations in the U.K., we intend to identify where targeted interventions could be implemented to improve the inequities as well inequalities faced by minority populations [26,27]. This qualitative study follows the Population, Interest, Context (PICo) format (Table 3).

## 2. Materials and Methods

The methodology of this systematic review follows the PRISMA-DTA guidelines for a systematic review and meta-analysis [28].

### 2.1. Eligibility Criteria

Table 3 shows the study characteristics that are eligible for review. The study design follows the PICo format for a qualitative systematic review. 

Population: This study investigated all adult patients eligible or having undergone diagnostic head or brain imaging techniques to identify the differences in access to different imaging techniques. We excluded studies on autism, mental health and biomarker screenings on blood samples to focus on structural and functional imaging techniques. Studies of neurodevelopmental disorders were also excluded as they did not test on an adult population. 

Interest: As this is an observational study to see if there are any health inequalities at the stage of diagnosis, we searched for cohort studies, systematic reviews and meta-analysis with primary data on diagnostic neuroimaging. Studies that had no relevant data in relation to access to diagnostic imaging on the head or brain were also excluded. Those that discussed other measures of cognitive screening as a measure of illnesses that are diagnosed with imaging techniques were also included to look at a quantitative measure of disease progression at diagnosis despite imaging not being mentioned in the study. 

Context: Although the NHS provides services that should be accessible to all patients in the U.K., wed looked into the time taken for diagnosis and disease progression at diagnosis to observe any differences in care based on socio-economic status that would affect morbidity and mortality rates of patients. All studies on health care providers with access to diagnostic imaging will be included in this study. This means that data on private practices will also be included as the patient socio-economic characteristics may vary. This systematic review focuses on complete studies published between 2010 and 2021 to limit the search to the most recent data to determine any inequalities in the current health care system. As the study was based in the U.K., papers that were non-English and published outside of the U.K. were excluded. This study also takes into account the use of Clinical Commissioning Groups (CCG), which were introduced to the NHS system in 2013 and are responsible for primary care within local areas [29]. Service provided for each area is dependent on the demand, which means that access to specialised services varies across the U.K. Therefore, the outcomes of this study will allow for targeted interventions to improve health systems for subpopulations [26].

### 2.2. Search

The search process was carried out on several dates from 10 January 2021 until the final search on 10 July 2021. Data were collected from the Ovid (MEDLINE), PubMed, and Web of Science databases using the following search terms combined:

1. clinical commissioning groups;
2. refer/referral/referred;
3. United Kingdom OR England OR Wales OR Scotland OR Northern Ireland OR Great Britain OR GB OR UK;
4. ethnic * or equal * or inequal * or Black Asian Minority Ethnic;
5. head or brain or neuro *;
6. not neurodevelopmental;
7. imaging or imag * or diagnostic imaging or diagnos *;
8. sense or sensory or sensation;

Table 4 details the line-by-line advanced searches with Boolean operators used for each database search. With the Ovid search terms, “.mp” was added to terms 3, 6 and 7 to ensure a multi-purpose search was carried out and these terms were included in the title and/or abstract of the article, refining the search further. “*” and “$” were used to expand the search for changes in suffixes of the word. The searches were limited to publication between January 2010 and February 2021 to gather the most recent articles that are a reflection of the health care system at the time this systematic review was conducted. The databases were also limited to English articles only, as stated in the eligibility criteria. 

Search number 3 included all research conducted in the U.K., search 2 explored diagnostic imaging techniques, search number 4 collected any data based on racial inequalities or socio-economic deprivation, as both are associated and often mentioned together, and search 5 refined the searches to look at neuro-related diseases or impaired function. 

**Figure 1:**
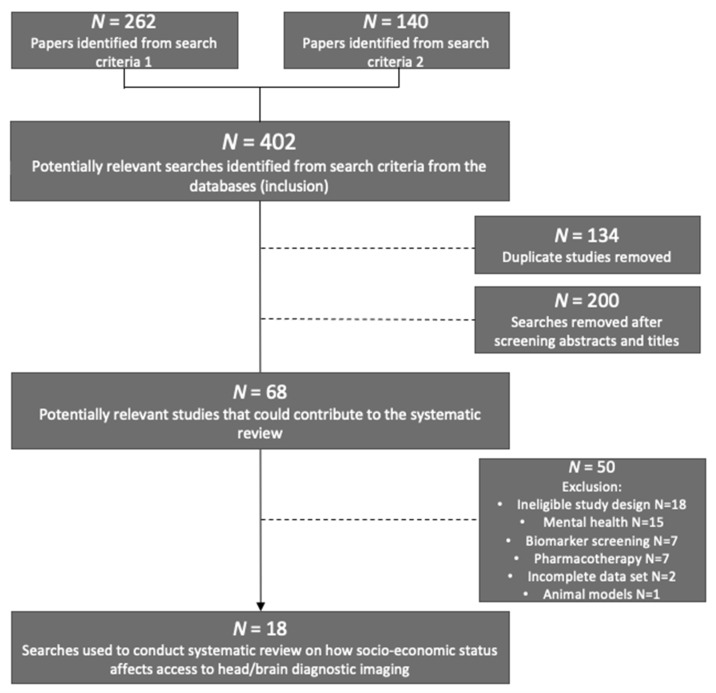
Flow chart to show the inclusion and exclusion of studies.

All searches were then exported from the databases to the Rayyan web-tool (https://rayyan.ai/) accessed on 7 April 2021. which detected duplicates between databases so that they could be removed. The inclusion and exclusion process is visually represented in Figure 1.

The dashed lines symbolise the removal of studies and the arrows represent the studies moving forward after each stage of the review. After the search terms were applied to the databases, a total number of 402 studies were suggested for this review. Duplicate studies were removed and abstracts and titles were screened and the remaining papers were kept for review. Those unsuitable were removed and 68 studies were reviewed for data eligibility. A total number of 18 studies were suitable as per the inclusion/exclusion criteria.

### 2.3. Study Selection

The first stage of selection included screening the titles and abstracts of articles to decide if the articles could be a relevant contribution to the research using the inclusion and exclusion criteria detailed in Table 2. Following this, the remaining studies were read and screened for any relevant data and findings on inequalities in access to diagnostic neuroimaging. While imaging type is important to the finding of this research, if it was not directly mentioned or measured, papers that reported other measures of disease progression and potential disparities with relevant findings were still included. This inclusion and exclusion criteria were based on the NICE guidelines for dementia, as it is assumed that data on diagnosis of these diseases would not be available without the use of diagnostic imaging [30]. 

Data from selected papers were collected by one reviewer and any discrepancies were discussed with two other independent reviewers (R. Zamani and M. Akrami). Where information was unclear, authors of the relevant article were contacted for clarification on data interpretation. Where data were missing for dates, it was assumed that data were collected close to the time of submission for peer review. 

The data outcomes in Table 4 display the data collected from each paper. The year of study is reflective of how accurate the study reflects the current health care system, as regulations and laws are regularly updated in order to improve patient experience and financial burden. Both type of imaging and location of study were reported as these have an effect on the study conclusion. The source of the data reported and interpreted was reported, as some studies use the same database and therefore, their findings may be similar for that reason.

### 2.4. Risk of Bias

There is a risk of bias from individual studies included in this systematic review with the selection of hospital locations and patient data collection. This was mitigated by assessing the risk of bias with individual journals by reporting the journal publication ranking using SCIMago Journal Ranking tool (https://www.scimagojr.com/), accessed on 3 March 2021. This ranked the journals into quartiles, 1 being of the highest quality and 4 being the lowest. This is a strong indication of the impact factor of a paper, as the more people that are subscribed to a journal, the higher likelihood of the research being seen and cited [31]. The journal ranking was used after the inclusion and exclusion criteria to assess the quality of the papers included. Papers with a “Q4” ranking were excluded and those in the “Q3” ranking were assessed based on the value of the information in the paper. 

## 3. Results

### 3.1. Search Results

The search of the PubMed, Ovid and Web of Science databases produced 339 articles, of which 62 were duplicates. After removing duplicates, 277 articles were screened for title and abstract using the eligibility criteria of inclusion and exclusion. This refined the studies to 51 potentially useful studies that could inform the research. Figure 1 outlines the number of sources eliminated after each review process and highlights the characteristics of those excluded from the review. Studies that did not use structural or functional imaging techniques, studies exploring inequalities in mental health care or pharmacotherapy, studies using animal models and ineligible study designs were excluded after review. After reading all articles available in full, only 18 studies were included in the final study selection with data relevant to the research question.

### 3.2. Study Characteristics 

**Figure 2:**
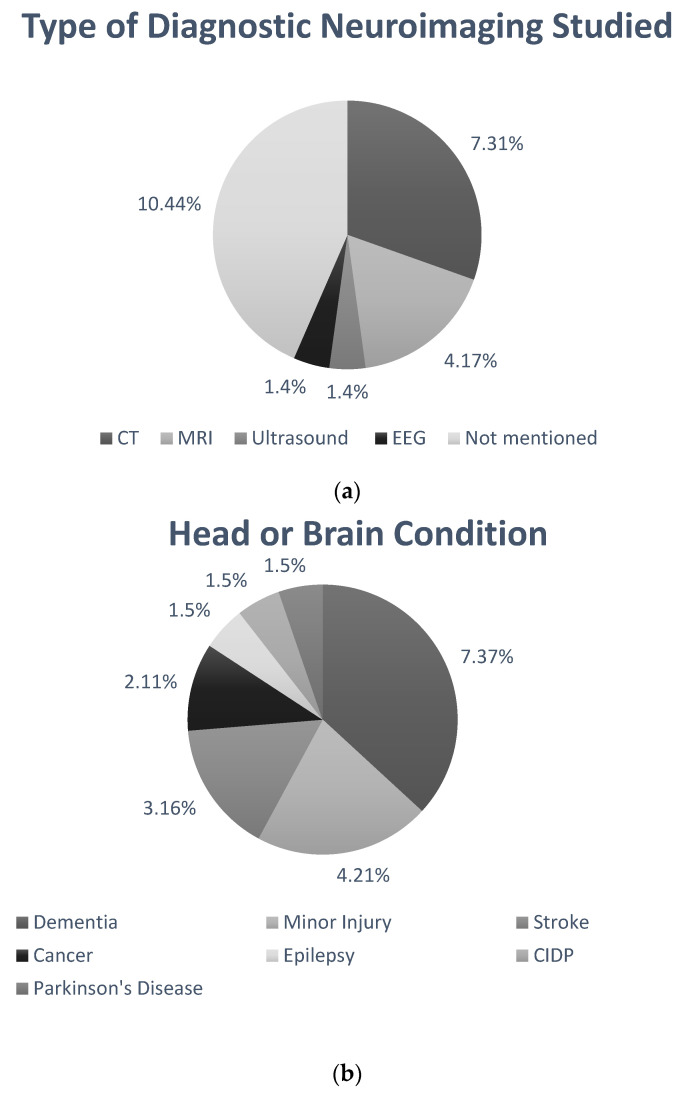
Pie charts representing the distribution of data collected. (**a**) This pie chart represents the distribution of data collected on neuroimaging techniques. Approximately 31% of studies reported on CT imaging techniques, 44% of studies did not mention imaging directly and ultrasound and EEG were only mentioned in one study each. This shows that there are not enough data available on ultrasound and EEG imaging and this should be considered for an area of further research. (**b**) This pie chart represents the distribution of data collected on neurological conditions. Approximately 37% of the studies presented data for dementia, showing that there is more research available on the health inequalities in dementia, while only one study reported on the accessibility of ultrasound and EEG imaging.

Eighteen papers were eligible for this systematic review. Their study characteristics and conclusions are detailed in Table 5. Figure 2a highlights the number of papers discussing each type of imaging. Approximately 31% of the studies discuss the use of CT. Figure 2b shows the distribution of studies included on each neurological condition. Approximately 38% of the studies (7/18) focused on the prevalence of dementia, while only one study discusses epilepsy. 

**Table 5:** Summary of results.

Condition	Reference	Year of Study	Journal Rank (SJR)	Type of Imaging	Location of Study	Data Collected	Source	Study Conclusion
**Dementia**	[32]	2014–2015	Q1	N/A	England	-HRQL/DEMQOL -Age -Sex -IMD -Ethnicity	Royal College of Psychiatrists national audit	-Association between HRQL and cognitive function—older, female, non-White and most deprived were more likely to have lower cognitive function at diagnosis
	[5]	2007–2015	Q1	N/A	London	-Ethnic background -Physical wellbeing -Functional scale -Medication -Dementia subtype	Clinical Record Interactive Search	-Vascular dementia is more common in black patients -Black patients were more likely to present with psychotic symptoms while South Asians were more likely to show depressive symptoms
	[33]	2008–2016	Q1	N/A	London	-Mental state -Age -Time of diagnosis -Ethnic background -Sex -IMD -Marital status	Clinical Record Interactive Search	-Association of cognitive score at diagnosis and ethnicity—mental state of at diagnosis was lower for Asian and Black patients compared to White patients -Association between age at diagnosis and ethnicity—Asian and Black patients were diagnosed about 4 years earlier in age compared to White patients
	[34]	2008	Q1	N/A	Greater Manchester	-Age -GP ownership -Referrals to secondary care -IMD	Primary Care Trusts and U.K. Department for Communities and Local Government	-Association between referral rates, practice socio-economic deprivation and number of GPs—higher rates of patients diagnosed with dementia in multi-handed practices in areas of greater socio-economic deprivation.
	[35]	2019	Q1	N/A	Northern Ireland, England, Wales and Scotland	-National guidelines -National polices -Recommendations -Migration references	National Alzheimer’s society, National Health and Social ministries, National Professional Societies for Geriatrics, Gerontology or Neurology	-From the U.K., only Northern Ireland refers to migration in the dementia guidelines -MMSE diagnostic tests are not culturally sensitive to language barriers -Language should factor into diagnosis of dementia
	[36]	2020	Q2	N/A	England and Wales	-Patient demographic -Patient referral -Availability of interpreters -Translation of resources -Cognitive assessment tools	Memory Assessment Service	-Translation services offered at MAS clinics vary around England and Wales -There are cognitive testing limitations with a patient language barrier
	[37]	2015–2016	Q1	N/A	London	-Ethnicity -Population diversity -Age	CCG	-This research has seen an improvement in engagement, awareness and help-seeking within minority communities when compared to previous research -There were fewer referrals than expected for some areas of London
**MHI**	[38]	2007–2008	Q2	CT	England and Wales	-Service provision for minor head injury -Access to CT	Hospital Episode Statistics And hospital emergency departments	-Most hospitals in England and Wales report following the NICE guideline and have unrestricted access to CT imaging -Observations and admissions of patients are mainly overseen by senior or specialist staff
	[39]	1990–2002	Q1	CT	North of England	-Patient identifiers -Date of birth -CT scans/type -Sex -Postcode	Radiology Information Systems (RIS)	-Association between deprivation score and head CT scan—people from more deprived areas required more head CT scans -Association between deprivation score and age of the scan—younger people from more deprived areas were needing more CT scans
	[40]	-	Q1	CT	England, Wales, Scotland, Northern Ireland	-GOS (patient outcome) -QALY -Cost -QoL	Harnen, S.E. et al., 2010	-It is more cost-effective to provide CT imaging for patients before discharge to avoid later cost of serve illness or disease. -For MHI, there should be unrestricted access to CT -Risk of cancer increases with age, giving an estimate of 0.002 for those at 75 years old
	[41]	2012	Q2	CT MRI	England and Wales	-MRI service hours -Radiographer’s training	NHS trusts	-Only 14% of NHS trusts have out-of-hours access to MRI imaging -1/3 trusts provided the basic MRI training for non-MRI radiographers
**Stroke**	[14]	2006–2009	Q1	CT MRI	England	-Brain image scanning -Age -Gender -Socio-economic deprivation -Comorbidity	NHS trust	-Only 35% of emergency stroke admissions receive a brain imaging scan immediately -16% do not receive imaging within period of hospital admission -Patient characteristics with a higher likely hood of receiving a scan: young, male, higher socio-economic status, fewer comorbidities
	[42]	2011	Q1	CT Ultra- sound MRI	England, Wales, Northern Ireland, Scotland	-Assessing the stroke-prevention and imaging services by: -volume of work, capacity, staff, type of imaging and timing of imaging	Royal College of Physicians Audit, Scottish stroke care, NHS improvement, Magnetic resonance National evaluation, Diagnostic Imaging Clinical Network, Stroke Collaborative	-CT was used as the main brain imaging technique in 84% clinics -51 clinics utilised MRI for stroke prevention -Waiting time for MRI after CT was about 1 month for 47% of the clinics -After a few days, TIA/minor stroke patients are unlikely to resent with signs of ischaemia with MRI, leading to a misdiagnosis and therefore, wasting the MRI. -Nurses with no specialist medial knowledge were in charge of medical assessment at 28% of centres
**Cancer**	[15]	1992–2012	Q3	CT MRI	Liverpool	-Cancer AJCC stage -IMD -Age	Aintree head and neck oncology database	-Association between deprivation and tumour presentation -Patients from more deprived areas had a higher rate of late tumour presentation at diagnosis -The introduction of a “two-week rule” in order to receive a faster diagnosis has seen improvement in the disease presentation at diagnosis; however, there is still a greater disease progression within more deprived patients
	[43]	2002–2005	Q1	N/A	Glasgow, Newcastle and London	-Sex -Age -Socio-demographic characteristics -Anthropometric measures -Smoking/alcohol consumption -Medical and dental history -Intake of selected foods	ARCAGE	-Association between gender and hospital admissions—greater inequalities for men observed -Association between education and hospital admissions—low education attainment is associated with risky behaviours –Those in lower socio-economic groups had higher hospital admissions
**Epilepsy**	[44]	-	Q2	EEG	England, Wales, Scotland, Northern Ireland	-Hospital procedure for CSE -Access/ usage of EEG monitoring -Neurologist staff available	NHS trust	-33% if the NHS trusts had access to continuous EEG monitoring -2/18 trusts with access to EEG used it as soon as convulsive refractory status epilepticus began
**CIDP**	[45]	2015–2019	Q1	-	Birmingham	-ONLS -Age -Gender -Pre-referral diagnosis	Specialist inflammatory neuropathy service	-68.3% of patients had an alternative pre-referral diagnosis -Underdiagnosis leads to mistreatment -Poor electrophysiological records show weakness in patient care
**Parkinson’s disease**	[46]	2007–2008	Q1	-	Milton Keynes	-Use of dopaminergic therapies -PDQ-39 -HADS -PD sleep scale	NHS	-9% of patients had never seen a neurologist, showing that there is not equal access to specialist services -Lower education was used as a predictor of PD
‘-’ = missing data								

This summary reports on year of study as a measure of how accurately reflects on the current health care system. The data are categorised into types of neurological condition: dementia, MHI, stroke, cancer and epilepsy. Refer to the list of abbreviations.

### 3.3. Risk of Bias 

Most of the studies (17/18) were published in journals with a ranking of Q2 or higher. Missing data on other types of imaging and neurological conditions mean that the focus of our study explores the inequalities in the use of CT, MRI, ultrasound and EEG in diagnosing dementia, MHI, stroke, cancer and epilepsy. 

### 3.4. Result of Individual Studies 

#### 3.4.1. Dementia

All (100%, 7/7) of the studies reporting on dementia use cognitive function as a measure of quantitative disease progression and three of these studies use Index of Multiple Deprivation (IMD) as a measure of deprivation. Tsamakis et al., 2021, reported that mean IMD scores were slightly higher for Black African (32.1; SD 8.6) and Caribbean (31.4; SD 9.0) patients, although this was not a significant difference between the IMD scores of White British patients (26.1; SD 11.4) [5]. T. Schmachtenberg et al., 2020, reported that only Northern Ireland’s legislations on dementia take ethnicity, cultural and religious differences into consideration for cognitive testing. It is suggested that Mini-Mental State Examination (MMSE) does not take into account language barriers. The legislation also highlighted the increased prevalence of vascular dementia in African, Caribbean and Asian communities [20]. This is supported by a study looking into the prevalence of dementia in BAME communities [5]. The study found that African and Caribbean patients had odds ratios of 1.96 (95% CL 1.56–2.49) and 1.65 (95% CL 1.93–1.75), respectively, when compared to White British patients. This study also reported that Black African (19.9%; 62/310) and Caribbean (14.4% 239/1661) and Irish (14.5%; 91/626) patients were the most likely to experience living condition problems, whilst South Asian patients were the least likely (5.8%; 21/364). 

Brown et al., 2021, reported that the number of ethnic minority referrals was higher in half of the clinics surveyed (4/8) in Greater London, which saw >50% of ethnic minority patient referrals [36]. This was representative of the population in Greater London, which is made up of 55.1% ethnic minorities. Multiple studies reported an earlier age of dementia diagnosis and lower cognition assessment scores at diagnosis in ethnic minority communities. Mukadam et al., 2019, reported that the mean MMSE scores were lower for Asian patients (−1.25; 95% CL-1.79—0.71: *n* = 642) and Black patients (−1.82; 95% CL −2.13—1.52: *n* = 2008) when compared to White patients [33]. The results showed that Asian patients were, on average, 4.27 (95% CL 3.61–4.92) years younger at diagnosis and Black patients were 3.70 years younger (95% CL 3.27–4.13) than White patients. A study reported that although there were higher rates of dementia diagnosed in areas of lower socio-economic status (Wald Chi-square = 123.7, *p* < 0.001), there was still an under diagnosis of patients as the rates of diagnosis were under the estimated prevalence, showing that, on average, 27 patients (95% CL 22–23) remain undiagnosed for a practice size of 5269 [34]. In addition to these factors, delays also occur due to a lack of referrals at primary care. The data presented by Cook et al., 2018, highlight that referrals for BAME patients are significantly lower [37]. BAME patients have a referral Odds Ratio of 0.9 (*p* < 0.0001) when compared to White British patients. 

#### 3.4.2. Minor Head Injury 

Pearce et al., 2012, reported that there was a significant association between the age of a patient and the Townsend deprivation quantile [39]. Younger patients from more deprived areas are receiving CT imaging (*p* < 0.0001). A higher proportion of patients from areas of higher deprivation had fewer scans; however, this trend was not significant (*p* > 0.05). An association between head and neck CT examination and deprivation was reported (*p* < 0.0001), showing that people of lower Townsend scores were more likely to receive imaging for issues within the head and neck. The trend in younger patients receiving CT imaging was more likely due to injury. Holmes et al., 2012, found that the mean cost of providing a CT scan for all patients with minor head injury (GBP 2928) is cheaper than the mean cost of discharging all patients aged 40 (GBP 3305) [40]. A similar beneficial trend is seen with the Quality Adjusted Life Year (QALY), as providing imaging to all patients aged 40 (18.6897) is greater than discharging all patients (18.6669). With patients aged 75, there is also a benefit seen in the mean cost analysis of providing a CT scan for all (GBP 1574) compared to discharging patients (GBP 1718). This evidence proves that it would be more cost-effective to provide patients with a form of neuroimaging to prevent further damage and cost despite the fact that it is not accessible. A study conducted on CT access within hospitals in relation to minor head injury reported that only 3.4% (6/174) of hospitals in their survey did not have unrestricted access to CT technology; however, most patient admissions required approval by a senior or specialist doctor (119/174; 68.3%) [26].

#### 3.4.3. Stroke 

Despite being a mandatory process after being admitted to hospital post-suspected-stroke, it is reported that in 2008/09, only 34.7% (25,452/7339) of patients received a CT or MRI scan immediately [14]. This study also reported that 59.0% (43,267/7339) of patients were seen within one day of admission and 84.3% (61,798/7339) of patients were given imaging within their stay at the hospital. It was found that there was association between socio-economic deprivation and likelihood of receiving a brain imaging scan (OR 0.94; 95% Cl 0.89–0.99, *p* < 0.05) when compared to the least deprived. Similarly, Hauptfleisch et al., 2013, reported that 15/107 (14%) had the required access to MRI, whilst extended weekday access and regular weekend availabilities were offered in 67 and 87 trusts (63% and 81%), respectively [41]. Further supporting this finding, Brazzelli et al., 2013, reported that 31% (35/114) of the clinics surveyed reported access to a stroke clinic every day, with an average of 35% (140,000/400,000) of the Transient Ischaemic Attack (TIA) and stroke patients being seen within 24 h of admission [42]. This survey also reported that CT imaging was the main brain imaging technique used in 84% (98/114) of the clinics and only 51% (58/114) used MRI for stroke assessments. It was reported that 22% of brain imaging results took more than 2 days to be returned to the stroke service, accounting for further delays in treatment. Moreover, 95% (95/100) of the clinics surveyed in this study reported the usage of Doppler ultrasound (DUS) for carotid/vertebral imaging, with 80% reporting that results were provided on the same day. The DUS exam was followed by a repeat exam (19%; 19/99), CT (60%; 60/99) or contrast MRI (41%; 41/99) as a confirmation of diagnosis. 

#### 3.4.4. Cancer 

Langton et al., 2019, found that there was an association between deprivation and tumour presentation at diagnosis [15]. The level of patients presenting with late stage 3 or 4 oral cancer decreased in areas of increased deprivation between the reported years 1992–2000 (56%; 95% CI 48.7–62.5%; 118/212) and 2001–2012 (47%; 95% CI 42.2–51.2%; 233/499). Whilst this difference was found to be a significant (Chi squared *p* < 0.03), inequality remains between the more deprived group and least deprived groups in 2001–2012 (42%; 95% CI 37.5–46.3%; 211/504). There was also found to be an association between the location of oral cancer and deprivation, as 56% (236/425) of patients presenting with tumours on the floor of the mouth were from more deprived areas. Conway et al., 2010, reported an association between education, gender and Upper Aerodigestive Tract Cancer (UATC) [43]. Those with a tertiary (university level or equivalent) level of education were less likely to be diagnosed with UATC in Glasgow (26.1%; 23), Manchester (33.3%; 49) and Newcastle (27.6%; 24). If patients had a secondary level of education, they would have greater chances of being diagnosed with UATC in Glasgow (73.9%; 65), Manchester (66.7%; 98) and Newcastle (72.4%; 63). It was also reported that in the British Isles, men had greater Odds Ratios for developing UATC at the lowest level of education when compared with those who had a tertiary level of education (19.88; 95% Cl 2.55,154.94, *p* < 0.01). 

#### 3.4.5. Epilepsy 

Patel et al., 2015, reported that 33% of U.K. NHS trusts surveyed in the study had continuous access to EEG monitoring, while only 3% (2/18) of the trusts utilised the imaging technique at early signs of Convulsive Refractory Status Epilepticus (CRSE) [44]. This was despite EEG being recognised as a fundamental practice by NICE [16]. 

#### 3.4.6. Chronic Inflammatory Demyelinating Polyneuropathy (CIDP)

Some 68.3% (41/60) patients were incorrectly diagnosed after initial consultation, which resulted in delays of treatments [45]. The average delay time for the final diagnosis of CIDP for patients was 21.3 months (range: 2–132 months). 

#### 3.4.7. Parkinson’s Disease 

This study concluded that there were no gender differences for referral rate of PD (*p* > 0.5) [46]; however, those who were older at GP consultation were less likely to have a specialist neurology consultation (*p* < 0.005). Moreover, 37.6% of diagnoses were made by GPs. Delays between the initial diagnosis and consultation were over a year.

## 4. Discussion

### 4.1. Dementia 

Some 37% of the studies account for data on socio-economic differences in dementia care, demonstrating that there are inequalities in the prevalence of disease amongst minority communities. Studies highlight that Black and Asian patients face a greater risk of being diagnosed with dementia. The data show that there is a higher occurrence of vascular dementia [5,33]. This can be explained by the increased risk of hypertension in these communities, which causes a reduced blood flow to the brain [47,48]. These studies also emphasised the threat of earlier onset disease. Asian patients were diagnosed at an average of 4.27 years earlier than White British patients and Black patients were diagnosed at an average of 3.70 years younger [5,33]. Earlier prevalence of disease could be attributed to other social determinants of health faced by minority communities, such as diet and education [49]. 

Although Mukadam et al., 2019, reported a difference of 3.70 years in Black patients, other literature estimates that patients of African and Caribbean descent are reported to show signs of dementia at an average of 7.8 years earlier than White British patients [50]. This could suggest that the data collected by the Clinical Record Interactive search were only representative of London, where the population of Black communities is greater; therefore, the health inequalities faced by communities have a different impact. However, this could also indicate why Black patients appear to have a lower cognitive score at diagnosis, signifying higher rates of disease progression. In addition to this, it could signify the lack of referral rates. This is because older literature indicates that there is a smaller ethnicity difference recorded when there are fewer referrals of BAME populations reported [37]. This could be an alternative suggestion for the difference in age at diagnosis reported within this study. 

Cognitive function recorded by Mini-Mental State Exams (MMSE) suggested that disease progression was significantly higher at diagnosis of dementia in Black patients (−1.82; 95% Cl −2.13 to −1.52) [33], proposing that there was a delay in receiving a diagnosis. This may be due to the differences in disease presentation between ethnicities. Black patients have reported higher rates of psychotic symptoms [5]. The misconceptions surrounding mental health in African communities may prevent patients and their families from seeking medical attention [51,52], Help-seeking, due to the stigma surrounding dementia, may also be an issue amongst South Asian [53] and Chinese communities [54], At diagnosis, ethnic minority patients are more likely to be in crisis and at a worse state of mental health, triggering help-seeking behaviour [5], again contributing to the lower cognitive scores recorded at diagnosis. 

Furthermore, the variation of symptoms could be misdiagnosed or ignored at primary care [55]. As local GP’s act as gate keepers to specialist services, they may allow their own bias, stereotyping and clinical uncertainty to affect their judgment [12]. Considering that referrals are dependent on the quality of primary care within one’s local area, this would suggest an explanation for the association between lower IMD scores and an increased prevalence of disease. The IMD scores collected also indicate that patients from Black African and Caribbean backgrounds live in more deprived areas then those who are White British (*p* < 0.001) [5]. The increased prevalence of disease supports the finding that there is poorer health in migrant communities living in areas of more deprivation [56].

The under-diagnosis of disease of BAME patients highlighted by Cook et al., 2018, is supported by the lack of referrals in areas of lower socio-economic status, highlighted by Connoly et al., 2011, due to the fewer GP practices in areas of lower SES [34,37]. Although the study on GP practices was in 2011, before CCG came into action, recent data have highlighted that there are fewer GP practices per 100,000 patients in more deprived areas [57].

While the use of MMSE allows for quantitative comparison of disease progression, the Northern Ireland Legislations have recognised that MMSE does not take language barriers and cultural differences into account. Therefore, it may not be an accurate measurement for cognition at diagnosis [35]. The addition of translators and varied testing materials could skew the results, favouring the notion that minority communities have lower cognitive scores at diagnosis. This insight into testing emphasises the important use of imaging for diagnosis, as there are no language barriers affecting the results.

Despite the universal use of diagnostic imaging, the need for interpreted materials is still vital for patient engagement and comfort. A study found that these services were inconsistent across Memory Assessment Service (MAS) clinics in England and Wales, which reflected on the patient experience [36]. Clinics with a diverse workforce have proved to be the most effective, as this increases cultural sensitivity towards the patients of ethnic minorities and means that the staff can act as translators where appropriate. These clinics are often in regions of the U.K. where there is a diverse population, suggesting that the staff and inclusion of patients is reflective of the MAS location. In the event that translators are not available or cancelled appointments, new appointments are scheduled. This would have an impact the timeliness of the diagnosis, and, therefore, indirectly affect the treatment outcome. This research is similar to the systematic review conducted in the U.S. on neurology services, which found that clinics with a diverse workforce are successful in engaging with minority communities [12]. Overall, there was no conclusive evidence of a lack of accessibility to diagnostic imaging itself; however, patient experience could be improved by exploring the possibility of mitigating language barriers. 

### 4.2. Minor Head Injury 

A study examining the use of CT scans in the North of England establish that there was a greater use of CT neuroimaging in young adults in the most deprived areas [39]. This is most likely associated with the increased risk of injury through lifestyle exposures. This includes occupational hazards causing an increased risk of accident from labour-intensive jobs [58]. 

The data used in the study were collected from public NHS hospitals. Hence, they may only provide an overview of essential uses of diagnostic imaging and not precautionary uses. CT imaging has been proven to be cost-effective; therefore, it is understandable that it would be the first use of imaging for MHI in public hospitals. Treating severe trauma as a result of delays may cost the NHS more in treatment [40]. However, utilisation of different imaging techniques may occur in the private health care sector due to radiation exposure; patients who have the choice of private care may opt for MRI scans that offer less radiation exposure. Therefore, it is possible that this study conducted in NHS hospitals only accounts for the increase in CT scans being performed on patients from higher deprivation levels. 

A limitation of studies using Townsend scores as a measure of deprivation is that they disregard distance as a factor in access to specialist imaging. Townsend scores are based on residential post-code. While this is a barrier in receiving diagnostic imaging and specialist care, it is an issue that may that also affect those living in rural areas of England, which are not considered as deprived areas [32,39]. A better indication of deprivation would be to use the Index of Multiple Deprivation as this includes a “health and disability” division. This would take occupation, income and education into consideration rather than just basing it on location. 

A study suggested that patients with lower IMD scores had fewer scans then those from less deprived areas [39]. This evidence suggests that differences in access to imaging could be a result of hospital workload or the judgment of health care workers. This is because offering liberal use of CT imaging is more beneficial and cost-effective [40].

### 4.3. Stroke

NICE guidelines for stroke care suggest that MRI imaging should be conducted on the same day of hospital admission to determine the pathology [59]. Any further delays in MRI would decrease the value of imaging, as TIA and minor strokes are unlikely to be detected after a few days of the incident. Therefore, usage would be inappropriate and not cost-effective. Despite this, it is reported that only 35% of patients were seen within 24 h of admission. Further delays were accounted for by the delivery of imaging results; 22% (25/114) of centres surveyed in this study reported that it took longer 2 days to receive patient results [42].

In addition to this finding, the study reported that CT was routinely used as the main from of TIA detection in 84% (98/114) of clinics surveyed [42]. This opposes the NICE guidelines, which state that CT should only be used if there is a doubt of TIA diagnosis [50]. Clinicians may opt for CT imaging, as it is cheaper; however, this may not be the most beneficial to patients [60]. In this case, those who can afford private care or have a higher level of education may request an MRI. Hence, education and income would act as a barrier to imaging services. There was no mention of the utilisation of ultrasound reported by the NICE guidelines, suggesting that it may not be a form of essential imaging. However, it is reported that 80% of clinics provided results the same day with this imaging technique [38], showing that its use is effective for quick diagnosis. 

Since there is limited use of out-of-hours services, increasing the availability may relieve the pressure on MRI clinics [41], meaning that the scans are more accessible when needed. Out-of-hours services are essential for meeting the targets of stroke care and prevention; it is suggested that patients receive a scan within the hour of admission into hospital [14]. Those who are young (*p* < 0.001), male (*p* < 0.012) and with a higher SES (*p* < 0.001) are more likely to be offered a CT or MRI scan [14], suggesting that there may inequalities in relation to access to diagnostic imaging for stroke care as it would be based on professional opinion. This study also saw a relation to patients with fewer comorbidities (*p* < 0.001) having a greater chance of imaging. Patients from ethnic minorities are more likely to have comorbidities [61]; therefore, they may receive fewer imaging opportunities. 

Both of these studies conducted on the utilisation of neuroimaging in stoke care were conducted via anonymous surveys, which meant that it is not possible to see if service is dependent on the location of clinics [41,42]. Further research should be conducted reporting the patient demographics receiving the additional or required diagnostic imaging for stoke prevention. This will allow for the comparison of patient characteristics that could cause concern for bias in the choice of the patient receiving scans. 

### 4.4. Cancer 

Both studies documenting the association between tumour presentation and deprivation reported that people from areas of greater deprivation were diagnosed more frequently with late stage 3 or 4 UATC [15,43]. Although the gap between the more deprived and least deprived has decreased since the introduction of the “two-week rule”, there are still a greater number of patients in low socio-economic areas being diagnosed with late stage oral cancer [15]. Prevalence of disease recorded was greater in in areas of lower IMD scores [15]. This may be due to the social determinants of health affecting overall lifestyle choices. It is suggested that this increased prevalence amongst those with lower education is due to lifestyle choices. Lower levels of education are associated with risky behaviours such as alcohol intake, smoking and insufficient hygiene [43,62]. 

The additional cost of dental treatments in the U.K. [63] may deter patients and reduce engagement at primary care. This may factor into delays in referral and diagnosis, meaning that patients present with later stage tumours. In addition, education surrounding oral hygiene and symptoms may deter patients from help-seeking [64]. Bird et al., 2020, provided evidence that oral gastrointestinal endoscopy was inefficient to diagnose and concluded an investigation of pharyngolaryngeal cancers as 42.3% of cases were not identified, while 23.5% of esophagogastric cases were misdiagnosed [65]. Effective diagnosis requires strong communication between specialties, which further provides evidence that there is a potential weakness at the referral stage. This means that patients are at risk of misdiagnosis if the GP does not refer them to the correct secondary care, or if there are delays at this stage. This study also proposes that inter-specialty referral should be increased to reduce risk of delays. 

### 4.5. Epilepsy 

The study assessing EEG stated that the lack of access to EEG monitoring for hospitals was a barrier to following the NICE guidelines [16,44]. They reported that few hospitals had the access to equipment, while even fewer utilised the imaging where required. This suggests that training should be an area for improvement. A study conducted in 2001 reported that only 63% (33/52) of hospitals had on-site access to EEG services, while 35% (18/52) had emergency services [66]. This supports the finding that there is a lack of access to imaging services [44]. However, there are no data available on patient characteristics in relation to access to service; therefore, further research should be conducted. Another study assessed the number of patients being misdiagnosed as a result of the lack of access to specialist services and physicians [67]. This highlighted that 26.1% of patients were misdiagnosed, and, as a result, mistreated, having possible socio-economic and health impacts. 

### 4.6. Chronic Inflammatory Demyelinating Polyneuropathy (CIDP)

Although CIDP is more common in men [17], this study could have included a greater diversity of patients, as only 31.6% of the patients were women [45]. This would allow for a better indication of how clinical representation factors into delays in diagnosis, as suggested in this study.

It is suggested that delays could also be a result of bias at referral or a lack of communication between specialist and secondary clinics. As this is an avoidable issue, it could be beneficial to increase awareness of symptoms of CIDP and the clinical presentation of disease in patients. The lack of public awareness could have an impact on the rate of misdiagnosis. 

Misdiagnoses were recognised after a hospital stay or further patient relapse, highlighting the vulnerable position patients are left in if they have the wrong diagnosis and no access to the required treatment. Delays in diagnosis could lead to axonal loss and limit treatment options for the patient. This, therefore, underlines the urgency of utilising imaging techniques such as MRI. Furthermore, this would be more reliable and cost-effective than depending on presentation of disease, which varies from patient to patient.

### 4.7. Parkinson’s Disease (PD)

This study identified that there are patterns in the data between delays and patient characteristics [46], such as age and education, showing that patient characteristics can affect diagnosis and referral. Treating patients before a diagnostic assessment can put them at risk of further harm, showing that risks are not the same for all patients even while being treated in a public health care system. It was also identified that patients not having access to specialist care from neurologists is in breach of the NICE guidelines for PD [18]. As identified by the authors of the study, further research is needed to see if these findings are replicated across other regions of the U.K. 

### 4.8. Inequalities in Access to Diagnostic Neuroimaging Facilities

Overall, the studies highlight that there is a delay in accessing neuroimaging facilities [14,33,34,37,39,43,46]. This is further emphasised by the lower cognitive scores in BAME patients diagnosed with dementia, indicating that there is an average ratio 1.41:1 of BAME patients having a lower MMSE score when compared to White patients [5,32,46]. MMSE is used as an indication of disease progression; therefore, a lower score at diagnosis suggests a delay in referral to neuroimaging services. It is suggested that the delays in referral are due to lack of GP referrals in more deprived areas and the delayed help-seeking behaviours in minority communities [34,37,51,52,53,54]. 

### 4.9. Strengths and Limitation 

The main strength of this study is that it compares the difference in socio-economic factors between all regions of the U.K., whereas other studies have focused on patient demographic differences as a whole. This addresses the inconsistencies in CCGs in local areas and the differences in patient access to services.

Limitations of this study include the lack of data available on health inequalities in diagnostic neurology. It is suggested that this should be an area for further research to see if these findings can be replicated with other neurological diseases or other neuroimaging techniques. It would be a research point of interest to investigate the accessibility of more expensive, specialist imaging equipment, as CT, MRI, ultrasound and EEG are routinely used. 

Patient numbers of BAME communities is lower in areas of the U.K. where BAME are underrepresented [32,36], meaning that it is difficult to identify a pattern with the data collected in this study in order to conclude that these patients face a significant delay in diagnosis. This could also be a factor of the lack of minority communities’ engagement in research [68].

## 5. Conclusions

**Figure 3:**
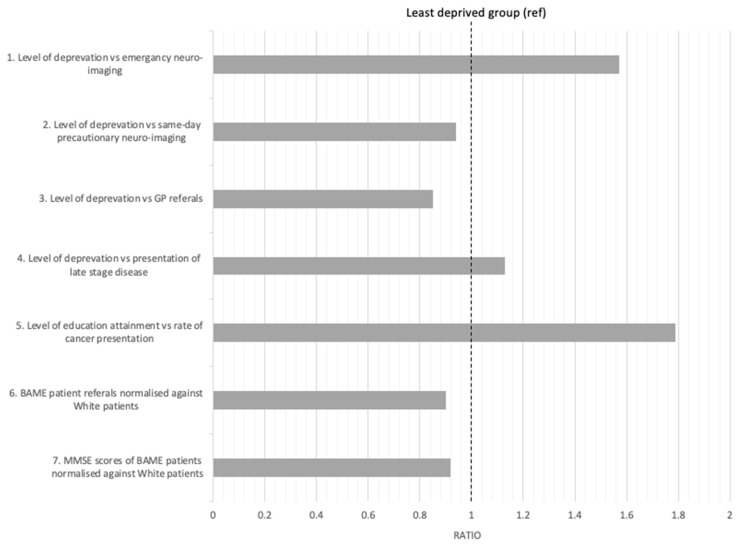
Analysis of some exemplar studies indicating the relationship between social deprivation factors and health care service provisions. (1) Level of deprivation measured in Townsend scores in North England for emergency CT neuroimaging [39]. Least deprived (*n* = 4078) is in the highest quantile and most deprived (*n* = 6395) is in the lowest quantile. A score above 1 highlights more patients in deprived areas accessing neuroimaging for head traumas. It is suggested the increased need is due to socio-economic factors such as risk-associated behaviours and manual labour occupations [39]. (2) High levels of deprivation measured by Carstair scores result in less precautionary same-day CT or MRI imaging of patients admitted with stroke management in English public hospitals [14]. The total number of scans in 2008/09 was 25452). A score below 1 indicates that fewer precautionary CT and MRI scans are taken for patients in lower quartiles of socio-economic deprivation at request of health care professionals. (3) High levels of deprivation measured in IMD scale indicate fewer numbers of referred cases of dementia by GP practices, taking Stockport (*n* = 293,594) and Manchester (*n* = 531,153) as examples of the least and most deprived regions in the area, respectively [34]. A score below 1 indicates an underdiagnosis of dementia patients in more deprived areas. (4) Level of deprivation specified by the Index of Multiple Deprivation’s (IMD) 2004 ranking against the patients presenting with late stage oral cancer in Liverpool [15] (most deprived (*n* = 351/711) against least deprived (*n* = 310/711)). A score above 1 indicates that more patients in the least deprived areas have a delayed diagnosis of oral cancer. (5) Level of education attainment as an index of socio-economic status is inversely proportional to cases of upper aerodigestive tract cancer in Glasgow, Manchester and Newcastle (*n* = 322) [43]. A score above 1 indicates how groups with a lower education attainment have higher rates of cancer presentation. (6) GP referrals of BAME patients to Memory Assessment Services (MAS) for dementia against White patients in London (*n* = 628,730) [37]. BAME patients are more socio-economically deprived and, therefore, this is a measure against White patients, who are representative of less deprived groups [56]. A score below 1 indicates an underrepresentation of BAME at MAS clinics due to the lack of referrals to access specialist diagnostic services. (7) Diagnostic Mini Mental State Examination scores as a measure of dementia progression at the time of diagnosis in BAME patients against White British patients in London (*n* = 13,233) [33]. A score below 1 indicates a delayed referral to MAS compared to White patients. Delayed referral highlights an inequality that may be due to delayed help-seeking behaviours in ethnic minority communities [51,52,53,54].

Our study has found that there is an association between the lack of access to diagnostic neuroimaging facilities and a patient’s socio-economic status from the evidence presented in the papers that were included in this study (Figure 3). Although there is a greater prevalence of disease associated with depravation, meaning that there is a greater number of patients of BAME communities seeking medical attention, there are also data to suggest that there is still under-representation in some areas of the U.K. This study shows how there is a higher disease progression of dementia and PD at diagnosis in patients of a lower SES, which could suggest delays in access to diagnostic treatment from GP referrals; therefore, this research area should be further explored. 

There is no conclusive evidence of disparities in relation to MRI, ultrasound or EEG imaging specifically from the available data, highlighting insufficient research in these areas. Data currently available on CT imaging has indicated that utilisation may not be the most effective in some cases. For example, MRI should be used instead of CT imaging for stroke management. This outcome supports the idea suggested in a recent BMJ article [26]. This state of poor quality of patient care could contribute to more mortality rates than the lack of access to services. Further research should be conducted on the appropriate use of imaging techniques to determine if this has an overall effect on morbidity and mortality rates of patients undergoing stroke care. In addition to this, the lack of data available on EEG imaging access for emergency service is a concern. Further research should identify the implications for lack of equipment in hospitals and possible suggestions to improve the services.

Although this study is exclusive to the U.K., it is clear that there are similar inequalities in Europe and America [12,43]. Therefore, a comparative study of health inequities in respect to access to diagnostic neuroimaging facilities should be conducted as a future research interest to highlight the global impact of health inequalities.

## Figures and Tables

**Table 3:** Inclusion and exclusion criteria.

	Inclusion Criteria	Exclusion Criteria
Population	Patients undergoing neuroimaging at: Memory Assessment Service clinics NHS hospitals Private hospitals in the U.K. Patients in need of neuroimaging services	Autism Mental health Studies on Children (<18 years of age)
Interest	Inequalities in access to diagnostic neuroimaging services	Biomarker detection in the blood
Context	Impact on neuroimaging services based on socio-economic status, age, ethnicity Studies conducted in England, Scotland, Northern Ireland and Wales	Studies without U.K.-based data
Study design	Cohort studies	Non-English studies Studies published before 2010

Our study follows the Population, Interest, Context (PICo) format.

**Table 4:** Line-by-line search advance search history.

		Ovid (MEDLINE)	PubMed	Web of Science
**Search terms**	1	(United Kingdom or England or Wales or Scotland or Northern Ireland or Great Britain or GB or UK) .mp. (access ^$^ or inequality ^$^ or equal ^$^) .af. limit 2 to abstracts (minority or ethni ^$^ or BAME or socio-economic) .af. (head or brain or neuro ^$^) .mp. limit 5 to abstracts limit 6 to yr = “2010-Current” (imaging or diagnos ^$^ or MRI or CT or CAT or PET or fMRI or ECG) .mp. limit 8 to abstracts 1 and 3 and 4 and 7 and 9	(United Kingdom or England or Wales or Scotland or Northern Ireland or Great Britain or GB or UK) AND (imaging or diagnos *) AND (access * or inequality * or equal *) AND (minority or ethni * or BAME or socio-economic) AND (head or brain or neuro *)	CU = (United Kingdom or England or Wales or Scotland or Northern Ireland or Great Britain or GB or UK) AND TS = (imaging or diagnos *) AND KP = (access * or inequality * or equal *) AND (minority or ethni * or BAME or socio-economic) AND KP = (head or brain or neuro *)
	2	(United Kingdom or England or Wales or Scotland or Northern Ireland or Great Britain or GB or UK) .mp. (access ^$^ or inequality ^$^ or equal ^$^) .af. limit 2 to abstracts (minority or ethni ^$^ or BAME or socio-economic) .af. (head or brain or neuro ^$^) .mp. limit 5 to abstracts limit 6 to yr = “2010-Current” (imaging or diagnos ^$^ or MRI or CT or CAT or PET or fMRI or ECG) .mp. limit 8 to abstracts 1 and 3 and 4 and 7 and 9 (“Clinical Commissioning Groups”) AND (refer *) .mp.	(ethnic * or equal * or inequal * or Black Asian Minority Ethnic) AND (referral or referred or refer) AND Imaging or imag * or diagnostic imaging or diagnos *) AND (United Kingdom or England or Wales or Scotland or Northern Ireland or Great Britain or GB or UK)) AND (head or brain or neuro *) AND (refer or referral or referred) NOT (neurodevelopment *)	(“Clinical Commissioning Groups”) AND (refer *)
Filters applied		From 2010 to 2021 English	Full text From 2010 to 2021	From 2010 to 2021 English

Af = all fields; mp = multi-purpose search; “*” and “^$^” indicate truncation, searching for variation of suffixes and prefixes. TS = topic; CU = country/region; KP = keyword plus. ‘AND’ and ‘OR’ Boolean operators were applied to combine searches, refining the database further. The same terms were used on each database for consistency. All searches were filtered for publication dates between 2010 and 2021. Where applicable, “English” language was selected.

## Data Availability

Not applicable.

## Abbreviations

AJCC	American Joint Committee on Cancer
ARCAGE	Alcohol-Related Cancer and Genetic Susceptibility in Europe
BAME	Black, Asian and Minority Ethnic
CCG	Clinical Commissioning Groups
CT	Computed Tomography
COVID-19	Coronavirus
CIDP	Chronic Inflammatory Demyelinating Polyneuropathy Doppler
DUS	Ultrasonography
EEG	Electroencephalography
GOS	Glasgow Outcome Score
GP	General Practitioner
HADS	Hospital Anxiety and Depression Scale
IMD	Index of Multiple Deprivation
MAS	Memory Assessment Service
MHI	Minor Head Injury
MRI	Magnetic Resonance Imaging
MMSE	Mini-Mental State Exam
NHS	National Health Service
NICE	National Institute for Health and Care Excellence
ONLS	Overall Neuropathy Limitation Score
OR	Odds Ratio
PD	Parkinson’s Disease
PDQ	Parkinson’s Disease Questionnaire
PET	Positron Emission Tomography
PICo	Population, Interest, Context
QALY	Quality Adjusted Life Years Gained
QoL	Quality of Life
SPECT	Single-Photon Emission Computed Tomography
SES	Socio-economic Status
TIA	Transient Ischaemic Attack

## References

[1] National Health Service (NHS) Progressive Neurological Condition Tool Kit. https://www.england.nhs.uk/rightcare/wp-content/uploads/sites/40/2019/08/progressive-neuro-toolkit.pdf.

[2] World Health Organization (WHO) Social Determinants of Health. https://www.who.int/westernpacific/health-topics/social-determinants-of-health.

[3] Community, Work, and School (CDC) (2020). Centers for Disease Control and Prevention. https://www.cdc.gov/coronavirus/2019-ncov/community/health-equity/race-ethnicity.html.

[4] Ahmadi-Abhari S., Guzman-Castillo M., Bandosz P., Shipley M.J., Terrera G.M., Singh-Manoux A., Kivimaki M., Steptoe A., Capewell S., O’Flaherty M. (2017). Temporal trend in dementia incidence since 2002 and projections for prevalence in England and Wales to 2040: Modelling study. BMJ.

[5] Tsamakis K., Gadelrab R., Wilson M., Bonnici-Mallia A.M., Hussain L., Perera G., Rizos E., Das-Munshi J., Stewart R., Mueller C. (2021). Dementia in people from ethnic minority backgrounds: Disability, functioning, and pharmacotherapy at the time of diagnosis. J. Am. Med. Dir. Assoc..

[6] Simpson L., Jivraj S., Warren J. (2016). The stability of ethnic identity in England and Wales 2001–2011. J. R. Stat. Soc. Ser. A (Stat. Soc.).

[7] Lievesley N. (2010). The Future Ageing of the Ethnic Minority Population of England and Wales.

[8] GBD 2016 Neurology Collaborators (2019). Global, regional and national burden of neurological disorders, 1990–2016: A systematic analysis for the Global Burden of Disease Study 2016. Lancet Neurol..

[9] Williams D.R., Priest N., Anderson N. (2016). Understanding associations between race, socioeconomic status and health: Patterns and prospects. Health Psychol..

[10] Kostopoulou O., Delaney B.C., Munro C.W. (2008). Diagnostic difficulty and error in primary care—A systematic review. Fam. Pr..

[11] Robinson L., Tang E., Taylor J.-P. (2015). Dementia: Timely diagnosis and early intervention. BMJ.

[12] Saadi A., Himmelstein D.U., Woolhandler S., Mejia N.I. (2017). Racial disparities in neurologic health care access and utilization in the United States. Neurology.

[13] Black Lives Matter About—Black Lives Matter. https://blacklivesmatter.com/about/.

[14] Lazzarino A.I., Palmer W., Bottle A., Aylin P. (2011). Inequalities in stroke patients’ management in English Public Hospitals: A survey on 200,000 patients. PLoS ONE.

[15] Langton S., Lowe D., Rogers S.N., Plüddemann A., Bankhead C. (2019). The impact of the UK ‘two-week rule’ on stage-on-diagnosis of oral cancer and the relationship to socio-economic inequalities. J. Cancer Policy.

[16] NICE Epilepsies: Diagnosis and Management, Guidance. NICE. https://www.nice.org.uk/guidance/cg137/chapter/1-Guidance#investigations.

[17] National Organization for Rare Disorders (NORD) Chronic Inflammatory Demyelinating Polyneuropathy. https://rarediseases.org/rare-diseases/chronic-inflammatory-demyelinating-polyneuropathy/.

[18] NICE Recommendations, Parkinson’s Disease in Adults, Guidance. NICE. https://www.nice.org.uk/guidance/ng71/chapter/Recommendations#diagnosing-parkinsons-disease.

[19] (2017). Tests for Diagnosing Dementia. https://www.nhs.uk/conditions/dementia/diagnosis-tests/.

[20] (2017). Mouth Cancer-Diagnosis. https://www.nhs.uk/conditions/mouth-cancer/diagnosis/.

[21] NICE (2007). Head injury NICE guideline. Head Injury.

[22] Xue G., Chen C., Lu Z.-L., Dong Q. (2010). Brain imaging techniques and their applications in decision-making research. Xin Li Xue Bao/Acta Psychol. Sin..

[23] Lenkov D.N., Volnova A.B., Pope A.R., Tsytsarev V. (2013). Advantages and limitations of brain imaging methods in the research of absence epilepsy in humans and animal models. J. Neurosci. Methods.

[24] Barlinn K., Alexandrov A.V. (2011). Vascular imaging in stroke: Comparative analysis. Neurotherapeutics.

[25] Mayo Clinic SPECT Scan. https://www.mayoclinic.org/tests-procedures/spect-scan/about/pac-20384925.

[26] Hirschhorn L.R., Magge H., Kiflie A. (2021). Aiming beyond equality to reach equity: The promise and challenge of quality improvement. BMJ.

[27] World Health Organization (WHO) Health Equity. https://www.who.int/westernpacific/health-topics/equity.

[28] Page M.J., Moher D., Bossuyt P.M., Boutron I., Hoffmann T.C., Mulrow C.D., Shamseer L., Tetzlaff J.M., Akl E.A., Brennan S.E. (2021). PRISMA 2020 explanation and elaboration: Updated guidance and exemplars for reporting systematic reviews. BMJ.

[29] National Health Service (NHS) About CCGs. NHS Clinical Commissioners. https://www.nhscc.org/ccgs/.

[30] NICE Overview. Dementia: Assessment, Management and Support for People Living with dementia and Their Carers. Guidance. NICE. https://www.nice.org.uk/guidance/ng97.

[31] Saha S., Saint S., Christakis D.A. (2003). Impact factor: A valid measure of journal quality?. J. Med. Libr. Assoc..

[32] Park M.H., Smith S.C., Neuburger J., Chrysanthaki T., Hendriks A.J., Black N. (2017). Sociodemographic characteristics, cognitive function, and health-related quality of life of patients referred to memory assessment services in England. Alzheimer Dis. Assoc. Disord..

[33] Mukadam N., Lewis G., Mueller C., Werbeloff N., Stewart R., Livingston G. (2019). Ethnic differences in cognition and age in people diagnosed with dementia: A study of electronic health records in two large mental healthcare providers. Int. J. Geriatr. Psychiatry.

[34] Connolly A., Gaehl E., Martin H., Morris J., Purandare N. (2011). Underdiagnosis of dementia in primary care: Variations in the observed prevalence and comparisons to the expected prevalence. Aging Ment. Health.

[35] Schmachtenberg T., Monsees J., Hoffmann W., van den Berg N., Stentzel U., Thyrian J.R. (2020). How is migration background con-sidered in the treatment and care of people? A comparison of national dementia care guidelines in Europe. BMC Public Health.

[36] Brown S., Livingston G., Mukadam N. (2021). A National Memory Clinic Survey to Assess Provision for People from Diverse Ethnic Backgrounds in England and Wales. Int. J. Environ. Res. Public Health.

[37] Cook L., Mukherjee S., McLachlan T., Shah R., Livingston G., Mukadam N. (2019). Parity of access to memory services in London for the BAME population: A cross-sectional study. Aging Ment. Health.

[38] Goodacre S.W., Pandor A., Pickering A. (2010). Management of isolated minor head injury in the UK. Emerg. Med. J..

[39] Pearce M.S., Salotti J.A., McHugh K., Kim K.P., Craft A.W., Lubin J., Ron E., Parker L. (2012). Socio-economic variation in CT scanning in Northern England, 1990–2002. BMC Health Serv. Res..

[40] Holmes M.W., Goodacre S., Stevenson M.D., Pandor A., Pickering A. (2012). The cost-effectiveness of diagnostic management strategies for adults with minor head injury. Injury.

[41] Hauptfleisch J., Meagher T.M., King D., de Heredia L.L., Hughes R.J. (2013). Out-of-hours MRI provision in the UK and models of service delivery. Clin. Radiol..

[42] Brazzelli M., Shuler K., Quayyum Z., Hadley D., Muir K., McNamee P., De Wilde J., Dennis M., Sandercock P., Wardlaw J.M. (2013). Clinical and imaging services for TIA and minor stroke: Results of two surveys of practice across the UK. BMJ Open.

[43] Conway D., McKinney P., McMahon A., Ahrens W., Schmeisser N., Benhamou S., Bouchardy C., Macfarlane G., Macfarlane T., Lagiou P. (2010). Socioeconomic factors associated with risk of upper aerodigestive tract cancer in Europe. Eur. J. Cancer.

[44] Patel M., Bagary M., McCorry D. (2015). The management of convulsive refractory status epilepticus in adults in the UK: No consistency in practice and little access to continuous EEG monitoring. Seizure-Eur. J. Epilepsy.

[45] Chaudhary U.J., Rajabally Y.A. (2021). Underdiagnosis and diagnostic delay in chronic inflammatory demyelinating polyneurop-athy. J. Neurol..

[46] Hu M.T., Butterworth R., Kumar V., Cooper J., Jones E., Catterall L., Ben-Shlomo Y. (2011). How common and what are the determinants of sub-optimal care for Parkinson’s disease patients: The Milton Keynes community study. Park. Relat. Disord..

[47] Lackland D.T. (2014). Racial differences in hypertension: Implications for high blood pressure management. Am. J. Med Sci..

[48] Alzheimer’s Society High Blood Pressure and Dementia|Alzheimer’s Society. https://www.alzheimers.org.uk/about-dementia/risk-factors-and-prevention/high-blood-pressure.

[49] Bell C.N., Thorpe R.J., LaVeist T.A. (2010). Race/ethnicity and hypertension: The role of social support. Am. J. Hypertens..

[50] Adelman S., Blanchard M., Rait G., Leavey G., Livingston G. (2011). Prevalence of dementia in African–Caribbean compared with UK-born White older people: Two-stage cross-sectional study. Br. J. Psychiatry.

[51] Campbell R.D., Long L.A. (2014). Culture as a social determinant of mental and behavioral health: A look at culturally shaped beliefs and their impact on help-seeking behaviors and service use patterns of black Americans with depression. Best Pr. Ment Health.

[52] Berwald S., Roche M., Adelman S., Mukadam N., Livingston G. (2016). Black african and caribbean british communities’ percep-tions of memory problems: “We don’t do dementia”. PLoS ONE.

[53] Giebel C.M., Worden A., Challis D., Jolley D., Bhui K.S., Lambat A., Kampanellou E., Purandare N. (2019). Age, memory loss and perceptions of dementia in South Asian ethnic minorities. Aging Ment. Health.

[54] Lam T.P., Sun K.S., Chan H.Y., Lau C.S., Lam K.F., Sanson-Fisher R. (2019). Perceptions of Chinese towards dementia in Hong Kong—Diagnosis, symptoms and impacts. Int. J. Environ. Res. Public Health.

[55] Piccardi C., Detollenaere J., Bussche P.V., Willems S. (2018). Social disparities in patient safety in primary care: A systematic review. Int. J. Equity Health.

[56] Ministry of Housing, Communities and Local Government (2020). UK Population by Ethnicity. https://www.ethnicity-facts-figures.service.gov.uk/uk-population-by-ethnicity/demographics/people-living-in-deprived-neighbourhoods/latest#overall-most-deprived-10-of-neighbourhoods-by-ethnicity.

[57] Public Health England (2021). Place-Based Approaches for Reducing Health Inequalities: Annexes. https://www.gov.uk/government/publications/health-inequalities-place-based-approaches-to-reduce-inequalities/place-based-approaches-for-reducing-health-inequalities-annexes.

[58] Cubbin C., Smith G.S. (2002). Socioeconomic inequalities in injury: Critical issues in design and analysis. Annu. Rev. Public Health.

[59] NICE Recommendations, Stroke and Transient Ischaemic Attack in over 16s: Diagnosis and Initial Management, Guidance. NICE. https://www.nice.org.uk/guidance/ng128/chapter/Recommendations#imaging-for-people-who-have-had-a-suspected-tia-or-acute-non-disabling-stroke.

[60] Rubin G.D. (2017). Costing in radiology and health care: Rationale, relativity, rudiments, and realities. Radiology.

[61] Cossrow N., Falkner B. (2004). Race/ethnic issues in obesity and obesity-related comorbidities. J. Clin. Endocrinol. Metab..

[62] Wu H., Zhang J., Zhou B. (2021). Toothbrushing frequency and gastric and upper aerodigestive tract cancer risk: A meta-analysis. Eur. J. Clin. Investig..

[63] National Health Service (NHS) (2020). Get Help with Dental Costs. https://www.nhs.uk/nhs-services/dentists/dental-costs/get-help-with-dental-costs/.

[64] El-Yousfi S., Jones K., White S., Marshman Z. (2019). A rapid review of barriers to oral healthcare for vulnerable people. Br. Dent. J..

[65] Bird J.H., Williams E.J., Heathcote K.J., Ayres L., De Zoysa N., King E.V., Parry S.D., Nouraei S.A.R. (2020). Interspecialty referral of oesophagogastric and pharyngolaryngeal cancers delays diagnosis and reduces patient survival: A matched case-control study. Clin. Otolaryngol..

[66] Ganesan K., Appleton R., Tedman B. (2006). EEG departments in great britain: A survey of practice. Seizure.

[67] Smith D., DeFalla B., Chadwick D. (1999). The misdiagnosis of epilepsy and the management of refractory epilepsy in a specialist clinic. QJM Int. J. Med..

[68] Alvarez R.A., Vasquez E., Mayorga C.C., Feaster D.J., Mitrani V.B. (2006). Increasing minority research participation through community organization outreach. West. J. Nurs. Res..

